# Effectiveness of BNT162b2 COVID-19 primary series vaccination in children aged 5–17 years in the United States: a cohort study

**DOI:** 10.1186/s12887-024-04756-5

**Published:** 2024-04-26

**Authors:** Rachel P. Ogilvie, J. Bradley Layton, Patricia C. Lloyd, Yixin Jiao, Djeneba Audrey Djibo, Hui Lee Wong, Joann F. Gruber, Ron Parambi, Jie Deng, Michael Miller, Jennifer Song, Lisa B. Weatherby, Lauren Peetluk, An-Chi Lo, Kathryn Matuska, Michael Wernecke, Christine L. Bui, Tainya C. Clarke, Sylvia Cho, Elizabeth J. Bell, Grace Yang, Kandace L. Amend, Richard A. Forshee, Steven A. Anderson, Cheryl N. McMahill-Walraven, Yoganand Chillarige, Mary S. Anthony, John D. Seeger, Azadeh Shoaibi

**Affiliations:** 1 Optum Epidemiology, Boston, MA USA; 2 RTI Health Solutions, 3040 East Cornwallis Rd, PO Box 12194, Research Triangle Park, NC 27709 USA; 3 US Food and Drug Administration, Silver Spring, MD USA; 4 Acumen, LLC, Burlingame, CA USA; 5 CVS Health, Blue Bell, PA USA; 6 Optum Serve, Falls Church, VA USA

**Keywords:** COVID-19 vaccines, Epidemiology, Vaccine effectiveness

## Abstract

**Background:**

COVID-19 vaccines are authorized for use in children in the United States; real-world assessment of vaccine effectiveness in children is needed. This study’s objective was to estimate the effectiveness of receiving a complete primary series of monovalent BNT162b2 (Pfizer-BioNTech) COVID-19 vaccine in US children.

**Methods:**

This cohort study identified children aged 5–17 years vaccinated with BNT162b2 matched with unvaccinated children. Participants and BNT162b2 vaccinations were identified in Optum and CVS Health insurance administrative claims databases linked with Immunization Information System (IIS) COVID-19 vaccination records from 16 US jurisdictions between December 11, 2020, and May 31, 2022 (end date varied by database and IIS). Vaccinated children were followed from their first BNT162b2 dose and matched to unvaccinated children on calendar date, US county of residence, and demographic and clinical factors. Censoring occurred if vaccinated children failed to receive a timely dose 2 or if unvaccinated children received any dose. Two COVID-19 outcome definitions were evaluated: COVID-19 diagnosis in any medical setting and COVID-19 diagnosis in hospitals/emergency departments (EDs). Propensity score-weighted hazard ratios (HRs) and 95% confidence intervals (CIs) were estimated with Cox proportional hazards models, and vaccine effectiveness (VE) was estimated as 1 minus HR. VE was estimated overall, within age subgroups, and within variant-specific eras. Sensitivity, negative control, and quantitative bias analyses evaluated various potential biases.

**Results:**

There were 453,655 eligible vaccinated children one-to-one matched to unvaccinated comparators (mean age 12 years; 50% female). COVID-19 hospitalizations/ED visits were rare in children, regardless of vaccination status (Optum, 41.2 per 10,000 person-years; CVS Health, 44.1 per 10,000 person-years). Overall, vaccination was associated with reduced incidence of any medically diagnosed COVID-19 (meta-analyzed VE = 38% [95% CI, 36–40%]) and hospital/ED–diagnosed COVID-19 (meta-analyzed VE = 61% [95% CI, 56–65%]). VE estimates were lowest among children 5–11 years and during the Omicron-variant era.

**Conclusions:**

Receipt of a complete BNT162b2 vaccine primary series was associated with overall reduced medically diagnosed COVID-19 and hospital/ED–diagnosed COVID-19 in children; observed VE estimates differed by age group and variant era.

**Registration:**

The study protocol was publicly posted on the BEST Initiative website (https://bestinitiative.org/wp-content/uploads/2022/03/C19-VX-Effectiveness-Protocol_2022_508.pdf).

**Supplementary Information:**

The online version contains supplementary material available at 10.1186/s12887-024-04756-5.

## Background

The burden of coronavirus disease 2019 (COVID-19) has been relatively mild among children [1], but COVID-19 hospitalizations increased among children as new SARS-CoV-2 variants circulated [2]. At the time of study execution, monovalent BNT162b2 (Pfizer-BioNTech’s messenger ribonucleic acid [mRNA] COVID-19 vaccine, Comirnaty^®^) was authorized for children aged 5–17 years. In preauthorization trials, BNT162b2 demonstrated immunogenicity and efficacy in preventing confirmed COVID-19 infection in children and adolescents [3, 4]. However, compared with adults, there are fewer available data on the real-world effectiveness of COVID-19 vaccination in US children.

The effectiveness of COVID-19 vaccines has been evaluated in different US geographic and healthcare settings, time periods, and age groups using an array of study designs and data sources [5–10]. As part of its continued surveillance of authorized vaccines, the US Food and Drug Administration (FDA) Biologics Effectiveness and Safety (BEST) Initiative evaluated the real-world effectiveness of monovalent BNT162b2 in US children using national insurance claims databases linked to immunization information system (IIS) vaccination records to improve vaccine capture and limit bias. The primary objective was to assess the effectiveness of receiving a complete primary series of monovalent BNT162b2 COVID-19 vaccination compared with being unvaccinated in preventing medically diagnosed COVID-19 and hospital/emergency department (ED)–diagnosed COVID-19 in children aged 5–17 years. Secondary objectives assessed vaccine effectiveness (VE) by age subgroup and variant era.

## Methods

### Population and data source

This cohort study used two commercial insurance administrative claims data sources: Optum pre-adjudicated claims and CVS Health adjudicated claims databases (Additional file, Supplemental Methods: Data Sources). To enhance vaccine administration capture, claims databases were supplemented with IIS COVID-19 vaccination records [11, 12] (Optum, 10 IIS from 10 US states; CVS Health, 11 IIS from 9 US states). Each data source’s study population was restricted to geographic areas of overlap between claims and IIS data.

The study period started on December 11, 2020, when BNT162b2 was authorized for ages 16–17 years, and ended at the latest complete IIS data for each jurisdiction. In Optum, end dates varied by IIS, ranging from September 30, 2021, to May 31, 2022; in CVS Health, the end date was March 31, 2022 for all IISs. BNT162b2 was the only COVID-19 vaccine authorized for children aged 5–17 years during the study period.

Vaccinated children were identified at their first recorded COVID-19 vaccine dose during the study period; children with non-BNT162b2, brand-unspecified, or unclassifiable COVID-19 vaccine records as the first observed dose were excluded. The date of dose 1 was assigned as Time 0 in vaccinated children (Additional file, eFigure 1). Unvaccinated children were one-to-one exact matched with replacement on calendar date and the following: age groups (5–11, 12–15, 16–17 years), sex, county of residence, immunocompromised status, pregnancy status, previous COVID-19 diagnosis, having a comorbidity increasing the risk of severe COVID-19 [13], and influenza vaccine receipt in the previous year. The calendar date of dose 1 for the vaccinated child was set as Time 0 for the matched unvaccinated child.

Children were eligible for inclusion on or after the date BNT162b2 was authorized for their age group (December 11, 2020, for ages 16–17; May 10, 2021, for ages 12–15; October 29, 2021, for ages 5–11). Vaccinated and matched unvaccinated children were required to meet the following inclusion criteria (Additional file, eFigure 1): at least 365 days of continuous medical and pharmacy coverage before Time 0 (including the date of the age group–specific vaccine authorization to ensure observation of all COVID-19 vaccine doses); be aged within the vaccine-authorized age range at Time 0; and reside within the catchment area of the linked claims-IIS data. Children were excluded if they had a procedure or diagnostic code for one of the following before Time 0: monoclonal antibody or convalescent plasma treatment (90 days); COVID-19 diagnosis (30 days); fever, nausea/vomiting, rash diagnosis (3 days); hospitalization or ED visit (3 days); or hospitalization or long-term care residence (on Time 0). Children selected as unvaccinated comparators could subsequently be vaccinated and enter the vaccinated group with a new Time 0.

### Exposure assessment

BNT162b2 doses were identified using brand-specific procedure codes for vaccine administration, pharmacy codes for vaccine products, or IIS vaccination records [11, 14, 15]. Dose number was inferred from the chronological order of observed doses within a child’s record. An unbranded dose or a dose of the same brand occurring within 3 days following another dose was considered a duplicate and was removed; if a dose for a different brand was received within 3 days, the brand of the dose was considered unclassifiable. Follow-up began for all vaccinated children at dose 1 (Time 0) regardless of future vaccine dose receipt.

Children were followed from Time 0 until the study outcome or censoring at the first occurrence of the following: last day of the IIS-specific study period; disenrollment from health plan; or deviation from the vaccine exposure assigned at Time 0 (Additional file, eFigure 2). For vaccinated children, deviation from vaccine exposure included receiving BNT162b2 dose 2 before day 17, failure to receive BNT162b2 dose 2 by day 42, receipt of any other brand of COVID-19 vaccine or an unclassifiable dose, or a third dose of BNT162b2 (Additional file, eFigure 2). For unvaccinated children, deviation included receiving a first dose of any COVID-19 vaccine.

### Outcome assessment

Two nested COVID-19 outcomes were evaluated separately: (1) medically diagnosed COVID-19, identified as a recorded COVID-19 diagnosis from hospital, ED, outpatient, or physician encounters; and (2) hospital/ED–diagnosed COVID-19 (as a proxy for more severe cases). Recorded COVID-19 diagnosis codes (ICD-10-CM U07.1) were identified in claims in any coding position. The recorded date of the first diagnosis was assigned as the outcome date.

### Covariates

Descriptive characteristics were measured on or before Time 0 (Additional file, eFigure 1) using enrollment, diagnosis, procedure, and pharmacy data [16]. Covariates included demographics, comorbidities, frailty indicators [17], healthcare utilization, and conditions potentially increasing risk of severe COVID-19 [13].

### Statistical analysis

Analyses were performed separately by data source. The distribution of characteristics by vaccination group were described with means, standard deviations (SD), medians, and first and third quartiles (Q1, Q3) for continuous variables, and counts and proportions for categorical variables. Covariate balance between vaccination groups was evaluated with absolute standardized differences [18].

Propensity scores were estimated with multivariable logistic regression models including all prespecified covariates and matching factors (eTable 1; eFigure 4). Stabilized inverse probability of treatment (sIPT) weights were estimated from propensity scores with truncation below the first percentile and above the 99th percentile of the propensity score distribution.

The cumulative incidence of each COVID-19 outcome was estimated in the sIPT-weighted vaccine exposure groups as 1 minus the Kaplan-Meier estimator [19]. Hazard ratios (HRs) for the association of vaccination status with COVID-19 outcomes were estimated using sIPT-weighted Cox proportional hazards models. The estimation of variance accounted for the presence of repeated individuals in the study cohorts; 95% confidence intervals (CIs) were estimated with robust sandwich variance estimators [20]. Cumulative incidence and HRs in the first 14 days of follow-up were evaluated as a negative control outcome (COVID-19 vaccines are not expected to produce an immune response until 10–14 days after vaccination) [21, 22].

Subgroup analyses were performed by age group (5–11, 12–15, 16–17 years) and by variant era (pre-Delta era, December 11, 2020–May 31, 2021; Delta era, June 1, 2021–December 24, 2021; Omicron era, December 25, 2021–end of data availability [23]). Variant era analyses were restricted to children with Time 0s within the era, with follow-up censored on the last day of the era. A post-hoc analysis evaluated the distribution of person-time spent in each variant era by age subgroup resulting from the staggered authorizations by age group.

Quantitative bias analyses [24, 25] estimated the impact of potential misclassification because of missing vaccine records by estimating corrected HRs accounting for a range of vaccine exposure sensitivities (Additional file, Supplemental Methods). A sensitivity analysis evaluated potential informative censoring by delaying censoring 7 days after receipt of a censoring vaccine dose. Additional sensitivity analyses evaluated the impact of potential outcome misclassification resulting from a recorded COVID-19 diagnosis on the same day as COVID-19 vaccination by removing Time 0 from follow-up and reordering censoring criteria so censoring for receipt of a censoring dose occurred first.

Data source–specific estimates were meta-analyzed using fixed-effects meta-analysis models. Analyses were performed with SAS version 9.4 (SAS Institute, Cary, NC). This surveillance activity was conducted as part of the FDA public health surveillance mandate and was not subject to Institutional Review Board oversight [16].

## Results

We identified 95,161 eligible children in Optum and 365,312 in CVS Health databases aged 5–‍17 years who received a first dose of BNT162b2 during the study period. In Optum, 97% of vaccinated children were exact matched to an unvaccinated child, leaving 92,338 in each vaccine exposure group (132,528 unique children); in CVS Health, 99% were matched, leaving 361,317 in each group (694,621 unique children) (Additional file, eFigure 3). In both groups in both data sources, the mean age was 12 years (SD 4 years), and 50% were female. The largest proportion resided in the Midwest in Optum (47%), and in the West in CVS Health (42%). Characteristics of the groups were well balanced on all measured characteristics in both data sources (selected characteristics in Table 1; complete characteristics in Additional file, eTable 1). The propensity score distributions in the matched vaccinated and unvaccinated children were similar, suggesting comparability between the groups before weighting (Additional file, eFigure 4).

**Table 1:** Selected characteristics of children vaccinated with BNT162b2 COVID-19 vaccine and matched unvaccinated children

Characteristic	Optum			CVS Health		
	Vaccinated with BNT162b2 *N* = 92,338	Matched^a^ Unvaccinated *N* = 92,338	ASD	Vaccinated with BNT162b2 *N* = 361,317	Matched^a^ Unvaccinated *N* = 361,317	ASD
**Characteristics at Time 0**						
Age, years						
Median (Q1, Q3)	13 (9, 15)	13 (9, 15)		12 (9, 15)	12 (9, 15)	0.00
Mean (SD)	12.11 (3.60)	12.08 (3.64)	0.01	11.76 (3.66)	11.71 (3.72)	0.01
Sex, N (%)						
Male	46,516 (50.38%)	46,516 (50.38%)	0.00	181,416 (50.21%)	181,416 (50.21%)	0.00
Female	45,822 (49.62%)	45,822 (49.62%)	0.00	179,901 (49.79%)	179,901 (49.79%)	0.00
Region, N (%)^b^						
Northeast	13,051 (14.13%)	13,051 (14.13%)	0.00	64,684 (17.90%)	64,684 (17.90%)	0.00
South	12,959 (14.03%)	12,959 (14.03%)	0.00	69,202 (19.15%)	69,202 (19.15%)	0.00
Midwest	43,572 (47.19%)	43,572 (47.19%)	0.00	75,498 (20.90%)	75,498 (20.90%)	0.00
West	22,756 (24.64%)	22,756 (24.64%)	0.00	151,933 (42.05%)	151,933 (42.05%)	0.00
**Characteristics in the 365 days before Time 0, N (%)**						
Influenza vaccination	47,759 (51.72%)	47,759 (51.72%)	0.00	187,076 (51.78%)	187,076 (51.78%)	0.00
Pneumococcal vaccination	87 (0.09%)	77 (0.08%)	0.00	360 (0.10%)	368 (0.10%)	0.00
Well-check/well-child preventive healthcare visit	63,290 (68.54%)	62,515 (67.70%)	0.02	253,176 (70.07%)	246,607 (68.25%)	0.04
**Characteristics assessed using all available data at or before Time 0, N (%)**						
Autoimmune disorders	1,067 (1.16%)	1,049 (1.14%)	0.00	4,273 (1.18%)	3,995 (1.11%)	0.01
Chronic lung diseases (e.g., asthma, COPD, cystic fibrosis, pulmonary embolism)	10,086 (10.92%)	10,081 (10.92%)	0.00	43,858 (12.14%)	44,283 (12.26%)	0.00
Dementia or other neurological conditions	3,110 (3.37%)	3,331 (3.61%)	0.01	12,075 (3.34%)	12,542 (3.47%)	0.01
Diabetes mellitus, type 1 or 2	436 (0.47%)	416 (0.45%)	0.00	1,480 (0.41%)	1,336 (0.37%)	0.01
Heart conditions (e.g., heart failure, coronary artery disease, arrhythmias)	3,037 (3.29%)	3,135 (3.40%)	0.01	12,537 (3.47%)	13,118 (3.63%)	0.01
Immunocompromised state	458 (0.50%)	458 (0.50%)	0.00	1,674 (0.46%)	1,674 (0.46%)	0.00
Mental health conditions	17,813 (19.29%)	16,871 (18.27%)	0.03	63,102 (17.46%)	58,900 (16.30%)	0.03
Obese or severely obese	7,750 (8.39%)	8,138 (8.81%)	0.01	35,224 (9.75%)	37,775 (10.45%)	0.02
At least one COVID-19 laboratory performed	36,002 (38.99%)	32,568 (35.27%)	0.08	174,485 (48.29%)	157,714 (43.65%)	0.09
COVID-19 diagnoses occurring outside of a hospital or emergency department	2,759 (2.99%)	2,751 (2.98%)	0.00	10,878 (3.01%)	10,812 (2.99%)	0.00
Hospitalization or emergency department–diagnosed COVID-19	116 (0.13%)	129 (0.14%)	0.00	514 (0.14%)	643 (0.18%)	0.01

ASD = absolute standardized difference; COPD = Chronic obstructive pulmonary disease; COVID-19 = coronavirus disease 2019; Q1, Q3 = first and third quartiles; SD = standard deviation; US = United States

^a^Matched children on calendar date, age group, sex, US county of residence, immunocompromised status, pregnancy status, history of COVID-19 diagnosis, presence of a comorbidity identified by the Centers for Disease Control and Prevention as increasing individuals’ risk of severe COVID-19, influenza vaccination in previous year

^b^County and state of residence were used for matching and propensity score models, but US census region is displayed in descriptive tables for brevity

**Fig. 1:**
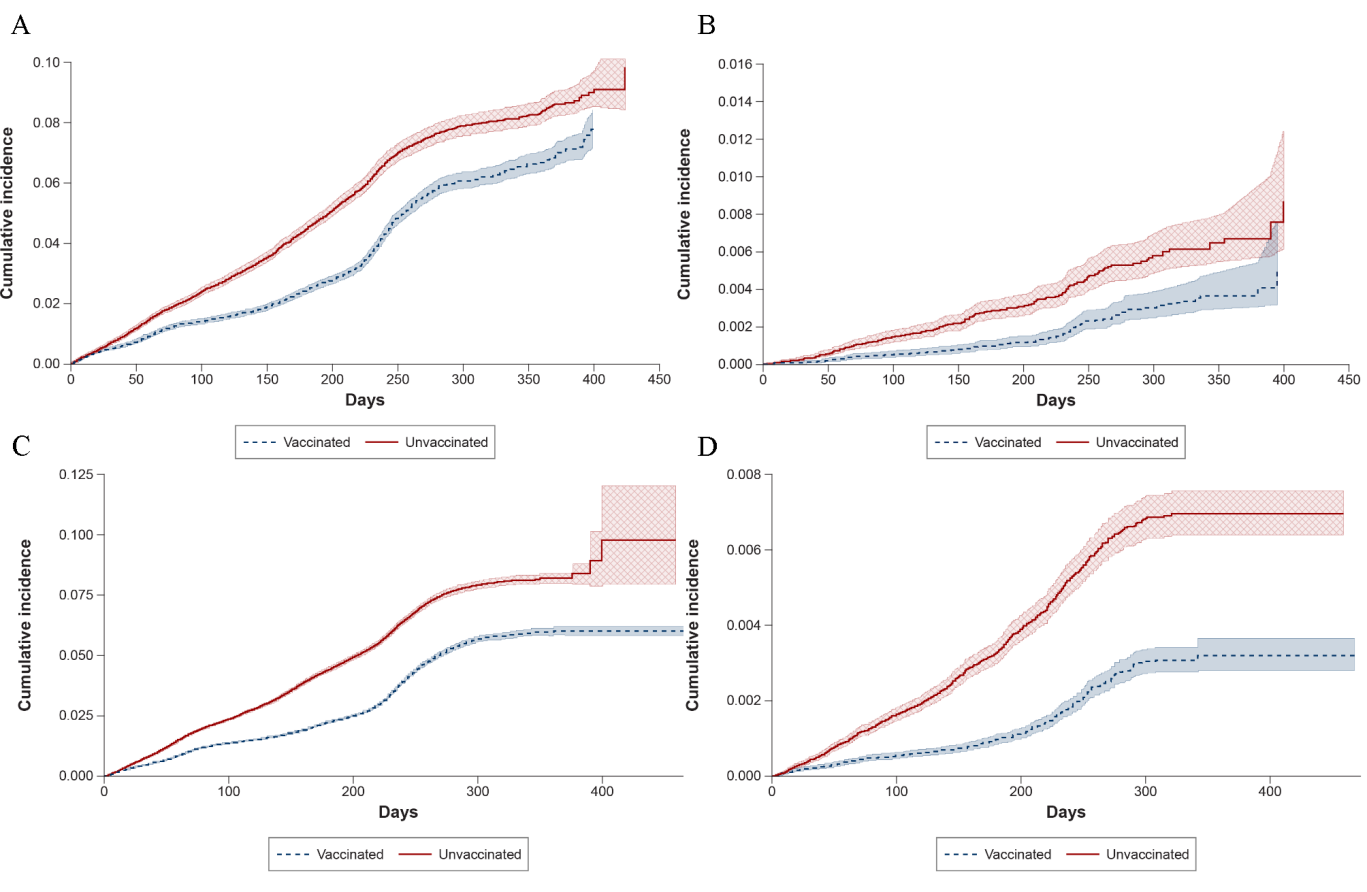
Cumulative Incidence of COVID-19 Outcomes in Children Receiving a Primary Series of BNT162b2 and Unvaccinated Comparators. (**A)**. Optum, Medically Diagnosed COVID-19. (**B**). Optum, Hospital/ED–diagnosed COVID-19. (**C**). CVS Health, Medically Diagnosed COVID-19. (**D**). CVS Health, Hospital/ED–diagnosed COVID-19. COVID-19 = coronavirus disease 2019; ED = emergency department.

**Fig. 2:**
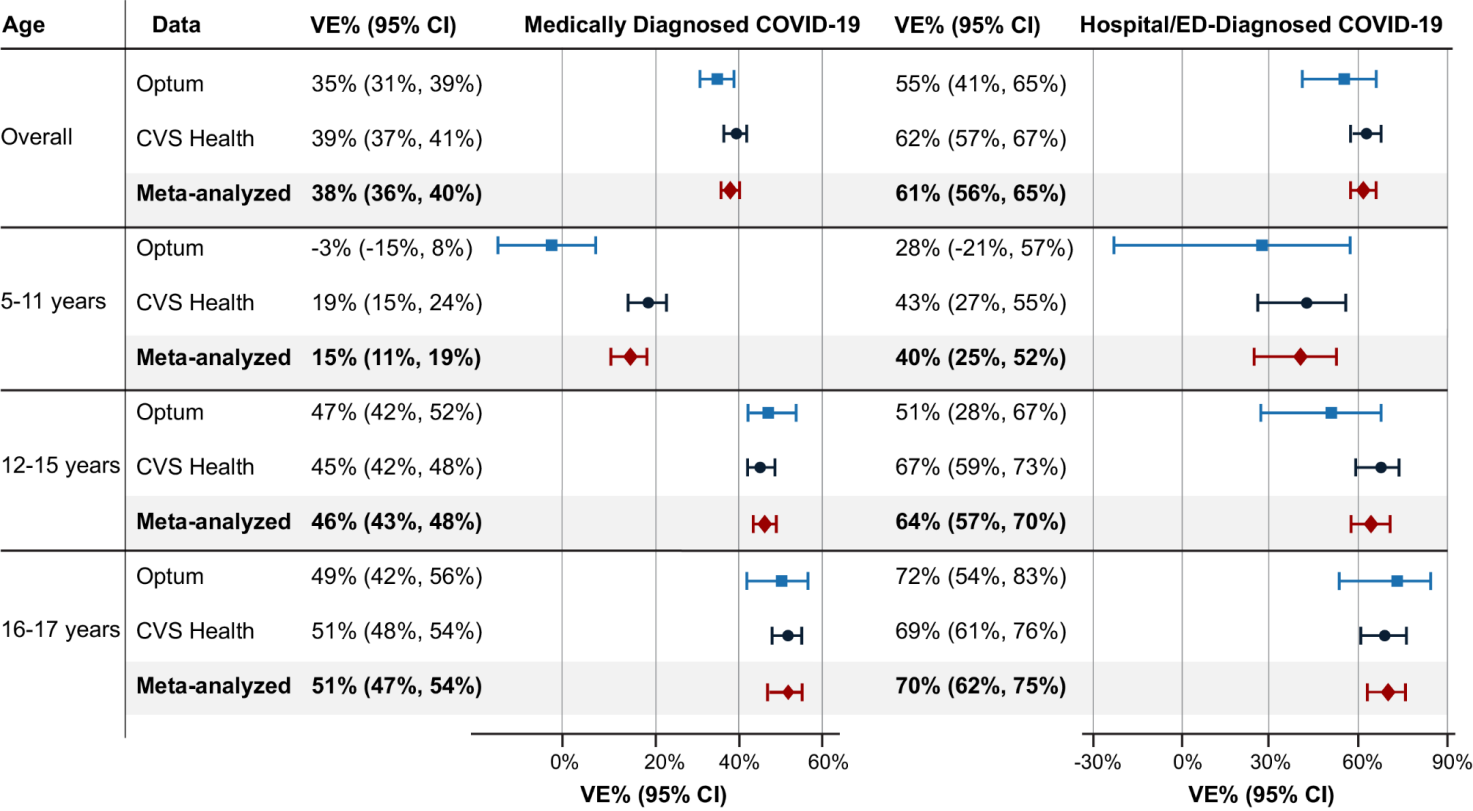
Effectiveness of a complete primary series of BNT162b2 compared with being unvaccinated. *Note*: Children were eligible for inclusion on or after the date BNT162b2 was authorized for their age group (December 11, 2020, for ages 16–17 years; May 10, 2021, for ages 12–15 years; October 29, 2021, for ages 5–11 years). CI = confidence interval; COVID-19 = coronavirus disease 2019; VE = vaccine effectiveness

The amount of follow-up time ranged across data sources and analyses, but the maximum follow-up time was 529 days in Optum and 468 days in CVS Health; the median follow-up tended to be longer for the vaccinated group in both data sources for each analysis (Additional file, eTable 2). The cumulative incidence of COVID-19 outcomes over time by vaccination group is shown in Fig. 1. Across all analyses in both data sources, the rates of hospital/ED–diagnosed COVID-19 cases were relatively small (Optum, 41.2 per 10,000 person-years; CVS Health, 44.1 per 10,000 person-years); approximately 94% of all observed COVID-19 diagnoses occurred outside of a hospital/ED setting (Additional file, eTable 3). For the main analyses, the risk of COVID-19 outcomes was higher in the unvaccinated than vaccinated groups throughout follow-up. The overall sIPT-weighted VE estimates for medically diagnosed COVID-19 were 35% (95% CI, 31–39%) in Optum and 39% (95% CI, 37–41%) in CVS Health (meta-analyzed VE = 38% [95% CI, 36–40%]). For hospital/ED–diagnosed COVID-19, VE estimates were 55% (95% CI, 41–65%) in Optum and 62% (95% CI, 57–67%) in CVS Health (meta-analyzed VE = 61% [95% CI, 56–65%]) (Fig. 2; Additional file, eTable 3). Using sensitivity estimates for the claims/IIS-based measures of COVID-19 vaccine exposure of 83% and 71% in Optum and 89% and 69% in CVS Health, quantitative bias analyses suggested that the observed VE estimates may underestimate the true VE by 2–13% (Additional file, eTable 4).

During the 14-day negative control period (Time 0 to day 13), the absolute risk of COVID-19 outcomes was low, and the absolute difference between the vaccinated and unvaccinated groups was small (Additional file, eFigure 5). However, estimated VEs during this period indicated potential associations of vaccination with medically diagnosed COVID-19: Optum VE = 15% (95% CI, − 1.7–28%); CVS Health VE = 25% (95% CI, 17–32%) (Additional file, eTable 5); negative control estimates for hospital/ED–diagnosed COVID-19 were imprecise as a result of having few cases. Post-hoc explorations of COVID-19 testing patterns during the negative control period in Optum suggested outcome misclassification caused by less frequent COVID-19 testing and diagnoses in the vaccinated group in the 3 to 4 days after vaccination (Additional file, eFigure 7).

When stratifying by age subgroup, VE estimates were generally high and similar across data sources for 16- to 17-year-olds (medically diagnosed COVID-19 meta-analyzed VE = 51% [95% CI, 47–54%]; hospital/ED–diagnosed COVID-19 meta-analyzed VE = 70% [95% CI, 62–75%]) and 12- to 15-year-olds (medically diagnosed COVID-19 meta-analyzed VE = 46% [95% CI, 43–48%]; hospital/ED–diagnosed COVID-19 meta-analyzed VE = 64% [95% CI, 57–70%]) (Fig. 2). In both data sources, VE estimates were lower in 5- to 11-year-olds. For 5- to 11-year-olds, the meta-analyzed VE for hospital/ED–diagnosed COVID-19 was 40% (95% CI, 25–52%); however, for medically diagnosed COVID-19, heterogeneity was observed across data sources (*p* < 0.01), with an Optum VE estimate of − 3% (95% CI, − 15–8%) and a CVS Health VE estimate of 19% (95% CI, 15–24%).

**Fig. 3:**
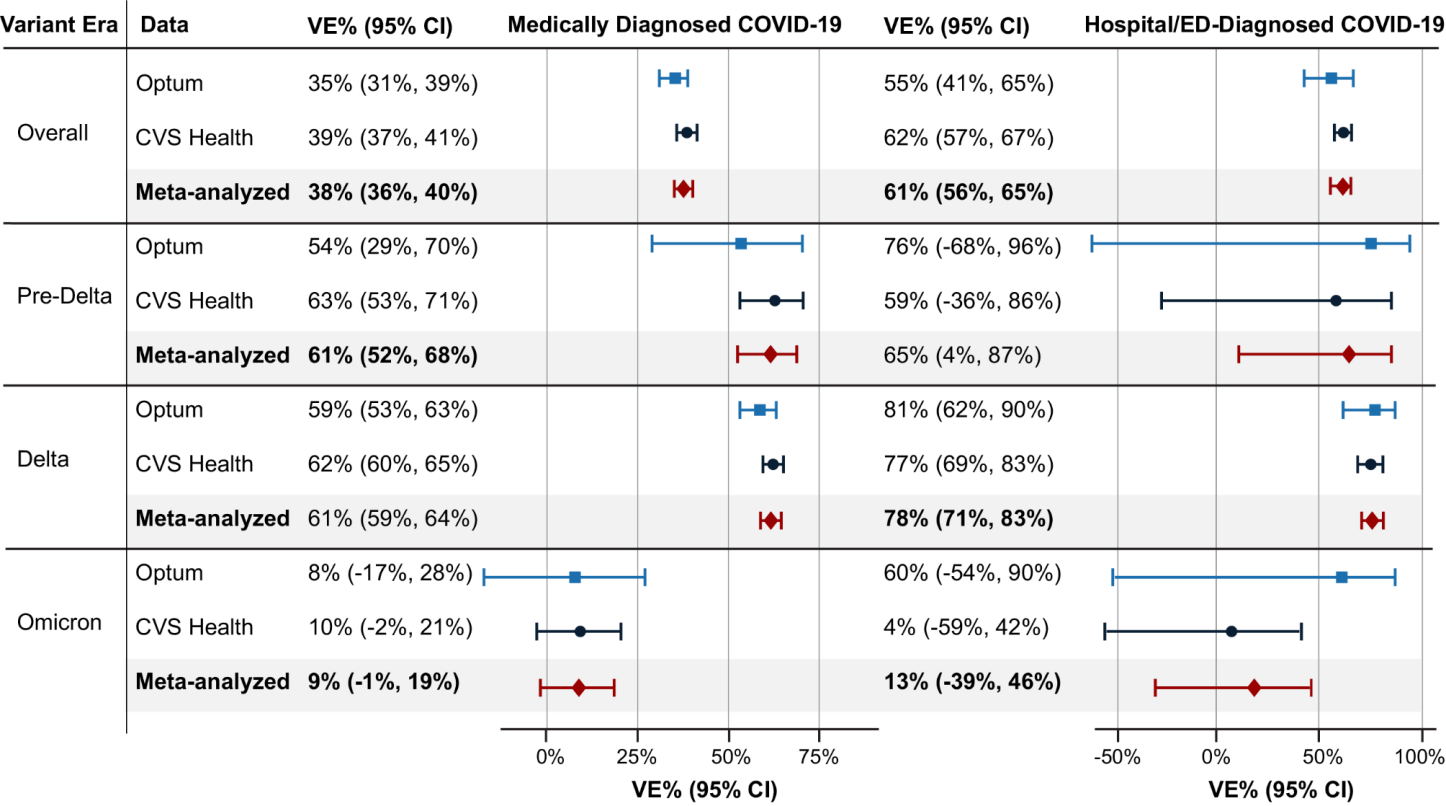
Effectiveness of Receiving a Complete Primary Series of BNT162b2 by Variant Era. *Note*: Pre-Delta era, December 11, 2020, through May 31, 2021; Delta era, June 1, 2021, through December 24, 2021; Omicron era, December 25, 2021, through the end of data availability. CI = confidence interval; COVID-19 = coronavirus disease 2019; VE = vaccine effectiveness

When evaluating VE by variant era, the largest sample sizes and follow-up were observed in the Delta era (Additional file, eTables 6 and 7). Variant era was highly correlated with age group, with the majority of person-time in the pre-Delta era coming from the 16- to 17-year-olds, the majority of Delta-era person-time from 12- to 15-year-olds, and the majority of Omicron-era person-time coming from 5- to 11-year-olds (Additional file, eTable 6). The VE estimates during the Omicron era against medically diagnosed COVID-19 (meta-analyzed VE = 9% [95% CI, − 1–19%]) and against hospital/ED–diagnosed COVID-19 (meta-analyzed VE = 13% [95% CI, − 39–46%]) were markedly lower than during the pre-Delta or Delta eras (Fig. 3). However, although there was no statistical heterogeneity between the source-specific Omicron-era VE estimates against hospital/ED–diagnosed COVID-19 (*p* = 0.24), there was a large range between the VE estimates: Optum VE = 60% [95% CI, − 54–90%]); CVS Health VE = 4% [95% CI, − 59–42%]) (Additional file, eTable 7). Results of sensitivity analyses were similar to those of the primary analyses (Additional file, eFigure 6).

## Discussion

In this large, real-world evaluation of the effectiveness of a complete primary series of the original monovalent BNT162b2 vaccine in children aged 5–17 years, lower rates of COVID-19 diagnoses were observed among children receiving a complete primary series of BNT162b2 compared with unvaccinated children, indicating that this vaccine is effective in routine care. VE was higher for hospital/ED–diagnosed COVID-19 than for any medically diagnosed COVID-19 and lower for children aged 5–11 years and during the Omicron era.

These observed VE estimates in children are generally lower than many VE estimates reported for the primary series of BNT162b2 in adults [26–29]. US children aged less than 16 were vaccinated relatively late in the pandemic after many months of potential COVID-19 exposures and infection, largely during the Delta-variant era. Many COVID-19 infections in children were relatively mild during the early pandemic [30, 31], and previous history of COVID-19 infection earlier in the pandemic before vaccination may have conveyed some level of natural immunity in both the vaccinated and unvaccinated groups, reducing the observed VE estimates.

Our study utilized two data sources, and the overall results of higher VE estimates for hospital/ED–diagnosed COVID-19 than for medically diagnosed COVID-19 across all age groups is consistent with the results of other studies [32, 33]. Additionally, many of the age and variant subgroup results were largely consistent across data sources. Children aged 5–11 years were the last age group receiving vaccine authorization during the study period, making it difficult to disentangle the effect of age group and variant era. Both data sources suggested lower VE in 5- to 11-year-olds and in the Omicron era—consistent with other studies [33]—but there were key differences in the magnitude of the VE estimates across data sources in these subgroups. Although calendar time and geography were balanced across vaccination groups within each data source (accounting for local differences in COVID-19 circulation and severity), the two data sources covered different geographic areas and time frames. For example, the end date for all IIS jurisdictions in CVS Health was March 31, 2022, but in Optum, the end dates varied from September 30, 2021 (before the beginning of the Omicron era) to May 31, 2022 (into the “second Omicron wave” starting in April/May 2022) [34].

This study has several strengths including a large sample size, inclusion of multiple US geographic regions, and combining vaccine administrative claims with IIS vaccine records. The linkage of claims to IIS data supplemented the vaccine exposure data and reduced vaccine exposure misclassification from vaccine doses not recorded in claims data. However, some vaccine administrations may still have been missed. The study used external estimates of vaccine coverage among individuals younger than 65 years to quantify potential residual exposure misclassification and applied quantitative bias analysis to correct VE estimates. Because younger children tended to be vaccinated later in the study period when mass vaccination clinics were less common, and younger children may have lower levels of vaccination compared with adults [35], the extent of exposure misclassification may be overestimated.

Because pandemic conditions varied widely across geographic areas and time periods, vaccinated and unvaccinated children were matched on calendar time and county of residence to account for these differences. The eligibility and matching criteria were designed to identify vaccinated and unvaccinated children who were eligible for vaccination on each calendar day, avoiding selection bias. Starting follow-up on Time 0 without considering future vaccination behaviors avoided immortal person-time bias [36].

This real-world study has limitations. This study did not analyze laboratory-confirmed COVID-19 status, so the study relied on recorded claims-based diagnoses of COVID-19. Although COVID-19 diagnosis codes have shown reasonable validity for hospitalized cases [37–42], many COVID-19 cases may never be formally diagnosed in a healthcare setting, and the dynamics of COVID-19 testing and diagnosis changed over time. In particular, Omicron-era symptom intensity was generally milder than previous eras, CDC and healthcare providers had more detailed self-care guidelines, and home tests were more widely available during the Omicron era, which likely reduced the incidence of medically diagnosed COVID-19, but perhaps had less of an impact on hospital/ED–diagnosed COVID-19. Although asymptomatic or mild COVID-19 cases may not be identified with our outcome definitions, hospitalizations for COVID-19 are a meaningful metric for public health surveillance [43].

Despite matching and propensity score weighting, residual and unmeasured confounding may remain. The negative control analysis suggested a potential difference between the exposure groups immediately after vaccination when vaccines are assumed to have no biologic effect. However, the post hoc negative control analysis demonstrated differential COVID-19 testing and diagnoses in the 3–4 days after Time 0, because recently vaccinated individuals may not seek COVID-19 testing [44]. This difference in testing and diagnosis behavior appeared to resolve after day 4, but longer term differences in healthcare–seeking behavior cannot be ruled out.

Because of the staged authorization of vaccines by age group, the study could not evaluate the primary series’ VE by variant era and age groups simultaneously (e.g., only children aged 16–‍17 years were authorized to be vaccinated until nearly the end of the pre-Delta era, and all the children aged 5–11 years were vaccinated during the Delta or Omicron eras). The smaller number of cases diagnosed in hospital/ED settings resulted in less precise estimates for some of the subgroup analyses. Because of the small number of immunocompromised individuals in this study (fewer than 0.5% in each data source), we were unable to evaluate VE in children who were immunocompromised.

## Conclusions

Receiving a complete primary series of the original monovalent BNT162b2 was associated with reduced COVID-19 incidence compared to being unvaccinated in the pediatric population. BNT162b2’s effectiveness was higher for hospital/ED–diagnosed COVID-19 than for any medically diagnosed COVID-19, and it was higher among children aged 12–17 years compared with 5–‍11 years. In the rapidly changing dynamics of the COVID-19 pandemic, additional real-world studies are needed to evaluate the effectiveness of COVID-19 vaccines as booster doses and additional vaccine brands become available for this population.

### Electronic supplementary material

Below is the link to the electronic supplementary material.

Supplementary Material 1

## Data Availability

The data that support the findings of this study are available from the respective data holders (Optum, CVS Health, and the contributing Immunization Information Systems) but restrictions apply to the availability of these data, which were used under license for the current study, and so are not publicly available. Data are however available from the authors upon reasonable request and with permission of the data partners. Please contact Bradley Layton (jblayton@rti.org).

## Declarations

### Author’s information

All authors and research partners for this work are collaborators within the FDA Biologics Effectiveness and Safety (BEST) initiative.

## Abbreviations

ASD: Absolute standardized difference
BEST Initiative: Biologics Effectiveness and Safety Initiative
CI: Confidence interval
COPD: Chronic obstructive pulmonary disease
COVID-19: Coronavirus disease 2019
ED: Emergency department
FDA: US Food and Drug Administration
HR: Hazard ratio
ICD-10-CM: International Classification of Diseases, 10th Edition, Clinical Modification
IIS: Immunization information system
Q1, Q3: First and third quartiles
SD: Standard deviation
sIPT: Stabilized inverse probability of treatment
US: United States
VE: Vaccine effectiveness
